# Silencing *DACH1* Promotes Esophageal Cancer Growth by Inhibiting TGF-β Signaling

**DOI:** 10.1371/journal.pone.0095509

**Published:** 2014-04-17

**Authors:** Liang Wu, James G. Herman, Malcolm V. Brock, Kongming Wu, Gaoping Mao, Wenji Yan, Yan Nie, Hao Liang, Qimin Zhan, Wen Li, Mingzhou Guo

**Affiliations:** 1 Department of Gastroenterology & Hepatology, Chinese PLA General Hospital, Beijing, China; 2 Sidney Kimmel Comprehensive Cancer Center, Johns Hopkins University, Baltimore, Maryland, United States of America; 3 Tongji Hospital, Tongji Medical College of Huazhong University of Science and Technology, Wuhan, China; 4 Department of Gastroenterology, General Air Force Hospital, Beijing, China; 5 Department of Gastroenterology & Hepatology, The Affiliated Hainan Hospital of the Chinese PLA General Hospital, Hai Tang wan, Sanya, China; 6 State Key Laboratory of Molecular Oncology, Cancer Institute and Hospital, Chinese Academy of Medical Sciences & Peking Union Medical College, Beijing, China; Peking University Cancer Hospital and Institute, China

## Abstract

Human Dachshund homologue 1 (*DACH1*) is a major component of the Retinal Determination Gene Network. Loss of DACH1 expression was found in breast, prostate, lung, endometrial, colorectal and hepatocellular carcinoma. To explore the expression, regulation and function of *DACH1* in human esophageal cancer, 11 esophageal cancer cell lines, 10 cases of normal esophageal mucosa, 51 cases of different grades of dysplasia and 104 cases of primary esophageal squamous cancer were employed. Methylation specific PCR, immunohistochemistry, western blot, flow cytometry, small interfering RNAs, colony formation techniques and xenograft mice model were used. We found that *DACH1* expression was regulated by promoter region hypermethylation in esophageal cancer cell lines. 18.8% (6 of 32) of grade 1, 42.1% (8 of 19) of grade 2 and grade 3 dysplasia (ED2,3), and 61.5% (64 of 104) of esophageal cancer were methylated, but no methylation was found in 10 cases of normal esophageal mucosa. The methylation was increased in progression tendency during esophageal carcinogenesis (*P*<0.01). *DACH1* methylation was associated with poor differentiation (*P*<0.05) and late tumor stage (*P*<0.05). Restoration of *DACH1* expression inhibited cell growth and activated TGF-β signaling in KYSE150 and KYSE510 cells. *DACH1* suppressed human esophageal cancer cell tumor growth in xenograft mice. In conclusion, *DACH1* is frequently methylated in human esophageal cancer and methylation of *DACH1* is involved in the early stage of esophageal carcinogenesis. DACH1 expression is regulated by promoter region hypermethylation. *DACH1* suppresses esophageal cancer growth by activating TGF-β signaling.

## Introduction

Esophageal cancer is the fifth most malignant disease and has been ranked as the fourth leading cause of cancer related deaths in China. [1] Esophageal squamous cell carcinoma (ESCC) is the predominant histological type of esophageal cancer, and accounts for approximately 90% of esophageal cancer cases in the northern and central China. [2] Despite the development of multimodal therapies, the 5 year overall survival remains below 20%. [3] The mechanisms of esophageal carcinogenesis remain unclear. Multiple genetic and epigenetic alterations were regarded as important factors for developing esophageal cancer [4]–[6].

Dachshund homolog 1 (*DACH1*), a major component of the Retinal Determination Gene Network, is widely expressed in epithelial cells. Reduction of DACH1 expression was associated with poor prognosis in breast, prostate, lung, endometrial, colorectal and hepatic cancer. [7]–[13] The expression of DACH1 was regulated by promoter region hypermethylation in endometrial, colorectal and hepatocellular cancer. [11]–[13] *DACH1* suppressed human hepatocellular carcinoma by activating TGF-β signaling. [13] While the epigenetic changes and the function of *DACH1* in human ESCC remain unclear. In this study, we mainly analyzed the epigenetic changes and the mechanism of DACH1 on esophageal carcinogenesis.

## Materials and Methods

### Ethics Statement

The study protocols were approved by the Ethics Committee of the Chinese PLA General Hospital (Permit Number: 20090701-015), and written informed consent was obtained from the participants. All procedures of animal research were approved by the Animal Ethics Committee of the Chinese PLA General Hospital (Permit Number: 2013-X8-40) and all efforts were made to minimize suffering. The study was carried out in accordance with the guidelines of the 1975 Declaration of Helsinki and was consistent with good clinical practice guidelines and local regulatory requirements.

### Primary Human Tissue Samples and Cell Lines

A total of 104 cases of primary esophageal squamous cell carcinoma were collected as fresh frozen tissue from Chinese PLA General Hospital. All samples were classified by TNM stage, including stage I (N = 5), stage II (N = 66), stage III (N = 32), and stage IV (N = 1). Fifty one cases of esophageal dysplasia were collected as paraffin-embedded samples, including 32 cases of grade 1 dysplasia (ED1), 11 cases of grade 2 dysplasia (ED2) and 8 cases of grade 3 dysplasia (ED3). Ten cases of normal esophageal mucosa (NE) were collected by biopsy under endoscopy from Chinese PLA General Hospital. Among 104 cancer samples, 30 cases of paraffin blocks were available with matched adjacent tissue. Eleven human ESCC cell lines (KYSE30, KYSE70, KYSE140, KYSE150, KYSE180, KYSE410, KYSE450, KYSE510, TE1, TE3 and TE8) were included in this study. All ESCC cell lines were described previously [14]–[17], and maintained in RPMI-1640 (Invitrogen) supplemented with 10% fetal bovine serum and antibiotics. The Ethics Committee of the Chinese PLA General Hospital approved this study (Permit Number: 20090701-015), and written informed consent was obtained before the collection of tissue sample and cell lines.

### 5-Aza-2′-deoxycytidine Treatment, RNA Isolation and Semi-quantitative Reverse Transcription-PCR

ESCC cell lines were split to low density (30% confluence) 12 hours before treatment. Cells were treated with 5-aza-2′-deoxycytidine (5-AZA) (Sigma-Aldrich) at a concentration of 2 µM in the growth medium, which was exchanged every 24 h for a total 96-h treatment. At the end of treatment course, cells were collected and total RNA was isolated by Trizol reagent (Invitrogen). Semi-quantitative reverse transcription-PCR (RT-PCR) was performed as described Previously [12].

### Bisulfite Modification, Methylation Specific PCR and Bisulfite Sequencing

Genomic DNA from ESCC cell lines and primary ESCC tissues were prepared by proteinase-K method. Methylation Specific PCR (MSP) and Bisulfite Sequencing (BSSQ) were performed as described previously. [18], [19] MSP primers and BSSQ primers [12] were designed according to genomic sequences around transcription start site in the CpG island of *DACH1* gene (GenBank NM_080759.4) promoter region and synthesized (BGI) to detect unmethylated (U) and methylated (M) alleles.

### Immunohistochemistry (IHC)

Immunohistochemical staining for DACH1 was performed on 4µm thick serial sections derived from formal dehyde-fixed paraffin blocks using antibody against DACH1 (1∶500 dilution, Proteintech) as described previously. [12] The staining intensity and extent of the stained area were graded according to the German semi-quantitative scoring system, which was also described previously [12].

### Plasmid Construction and Transfection

The *DACH1* expression vector was a gift from Dr. Cvekl. The *DACH1* expression vector and the Smad-binding elements (SBE)-4 Luc reporter plasmid were described previously. [20] *DACH1* was also subcloned into plenti6-GFP vector. Shuttle vector constructs and the ViraPower Packaging Mix were cotransfected 293FT cells to obtain lentivirus according to the manufacturer’s protocol (Invitrogen). Lentivirus was then added to KYSE510 and KYSE150 cells, and sceened by Blasticidin (5 µg/ml, Invitrogen) to generate *DACH1* stably expressed cells. Lipofectamine 2000 (Invitrogen) was used for plasmid transfection. All constructs were confirmed by sequencing.

### Cell Viability Assay

*DACH1* stably expressed cells and unexpressed control were seeded onto 96-well plates (3×10^3^ cells/well), and the cell viability was measured daily for 96 hours using the Cell Counting Kit-8 (Dojindo Laboratorie) according to instructions of the manufacturer. The results were plotted as means ± SD. All assays were performed in triplicate and repeated for three times.

### Colony Formation Assay

*DACH1* stably expressed cells and unexpressed control (5×10^2^ cells/well) were plated in 2 ml complete growth medium. The medium and reagents were changed once at 72 hours. After 2 weeks incubation, cells were fixed with 75% ethanol for 30 minutes and stained with 0.2% crystal violet (Beyotime) for 20 minutes and counting. For each experiment, the colony formation assay was performed three times.

### Flow Cytometry Analysis

For cell cycle analysis, the Cell Cycle Detection Kit (KeyGen Biotech) was used according to manufacturer’s instructions. Each sample was analyzed by flow cytometry with a FACScan Flow Cytometer (Becton-Dickinson) using a 488 nm laser. Histograms were analyzed for cell cycle compartments using ModFit version 2.0 (Verity Software House). For apoptosis analysis, the Annexin V-FITC Apoptosis Detection Kit (KeyGen Biotech) was conducted according to manufacturer’s instructions.

### DACH1 Knock down by siRNA

Four selected small interfering RNAs (siRNA) targeting *DACH1* and RNAi Negative Control Duplex were used in this study, and the sequences were described previously. [12] RNAi oligonucleotide or RNAi Negative Control Duplex (GenePharma) was transfected into KYSE140 cells using Lipofectamine RNAiMAX Reagent (Invitrogen), according to manufacturer’s instructions.

### Dual-luciferase Reporter Assay

KYSE510 and KYSE150 cells (3×10^3^) were seeded onto 96-well plates, incubated for 24 hours and transfected with an appropriate combination of the reporter, expression plasmids and control vector, including SBE4-luc reporter plasmid (20 ng/well), pRL-TK control vector (2 ng/well) as an internal control reporter, and increasing amounts of *DACH1* exprssion plasmid. Cells were serum-starved for 36 hours and then stimulated with or without TGF-β1 (Peprotech) for 12 hours before luciferase assay. Relative luciferase activities were measured with the Dual Luciferase Reporter Assay system (Promega) according to the manufacturer’s instructions. For each experiment, the luciferase reporter assay was performed three times.

### Protein Preparation and Western Blot Analysis

Protein isolation and western blot were performed as described previously. [12] Primary antibodies were as following: DACH1 (Proteintech); c-Myc, p21, phospho-Smad3, cyclinD1 (Cell signaling Technology); CDK4 (Affinity); phospho-Smad2 (Millipore); cyclinE1, CDK2, Smad2 and Smad3 (Bioworld Technology); β-Actin (Beyotime). The blots were visualized using enhanced chemiluminescence (Pierce Bioscience).

### *In vivo* Tumorigenicity

*DACH1* stably expressed KYSE510 cells and unexpressed control (4×10^6^ cells in 0.2 ml phosphate-buffered saline) was subcutaneously injected into the dorsal flank of 5-week-old female BABL/c nude mice respectively; each group included 5 mice. The tumor size was measured every 5 days for 4 weeks since 5 days after implantation, and the tumor volume was determined with the following formula: tumor volume (mm^3^) = [length (mm)]×[width (mm)]^2/^2. All procedures were approved by the Animal Ethics Committee of the Chinese PLA General Hospital (Permit Number: 2013-X8-40). All efforts were made to minimize suffering.

### Statistical Analysis

Data collected from multiple independent experiments were presented as the mean ± SEM, and analyzed using the Student’s *t* test. DACH1 expression level between esophageal cancer and matched adjacent tissue samples were compared using the Wilcoxon signed-rank test. Spearman’s rank correlation coefficient was calculated for the evaluation of the correlation between expression and methylation of DACH1. The relationship between clinicopathologic characteristics and DACH1 methylation status were analyzed using chi-square test. A *p* value of less than 0.05 was considered statistical significance. All statistical analyses were performed using SPSS 15.0 software.

## Results

### DACH1 Expression is down Regulated by Promoter Region Hypermethylation in ESCC Cell Lines

**Figure 1:**
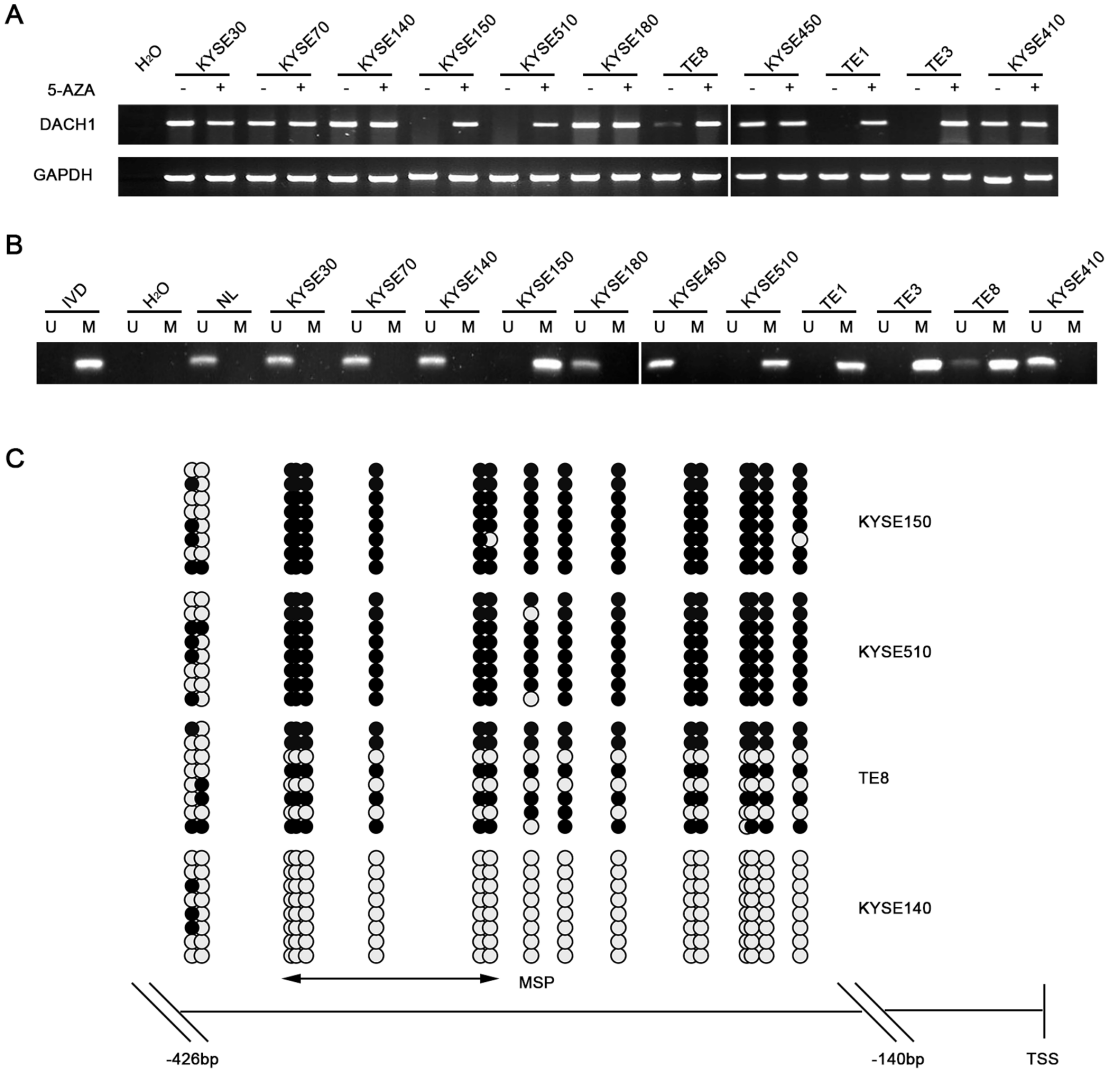
Representative results of *DACH1* expression and methylation in esophageal cancer cells. (A) *DACH1* expression level detected by RT-PCR in esophageal cancer cell lines. (B) Methylation status in promoter region; IVD: *in vitro* methylated DNA, used as methylation control; NL: normal blood lymphocyte DNA, used as unmethylation control; U: unmethylated alleles; M: methylated alleles. (C) BSSQ of *DACH1* promoter region (−426 bp to −140 bp) in KYSE150, KYSE510, TE8 and KYSE140 cells; double-headed arrow: MSP PCR product, spanning 130 bp. Filled circles: methylated CpG sites; open circles: unmethylated CpG sites.

DACH1 expression was regulated by promoter region hypermethylation in human colorectal and hepatocellular carcinoma. [12], [13] To explore the regulation of *DACH1* in ESCC, the expression and the methylation status of *DACH1* were detected by RT-PCR and MSP in esophageal cancer cell lines. As shown in figure 1A, Loss of DACH1 expression was found in KYSE150, KYSE510, TE1 and TE3 cells, reduced DACH1 expression was appeared in TE8 cells, and expression of DACH1 was detected in KYSE30, KYSE70, KYSE140, KYSE180, KYSE450 and KYSE410 cells. MSP results were shown in figure 1B. *DACH1* was completely methylated in KYSE150, KYSE510, TE1 and TE3 cells, partially in TE8, and unmethylated in KYSE30, KYSE70, KYSE140, KYSE180, KYSE450 and KYSE410 cells. MSP results were further validated by bisulfite sequencing (BSSQ) in KYSE150, KYSE510, TE8 and KYSE140 cells (Fig. 1C). Above results indicate that promoter region hypermethylation is correlated with loss/reduction of *DACH1* expression. Re-expression/increasing expression of *DACH1* was induced by 5-aza-2′-deoxycytidine (5-AZA) in *DACH1* methylated esophageal cancer cell lines (KYSE150, KYSE510,TE8, Fig. 1A). These data suggest that *DACH1* expression is regulated by promoter region methylation in ESCC cell lines.

### DACH1 is Frequently Methylated in Human Esophageal Cancer

**Figure 2:**
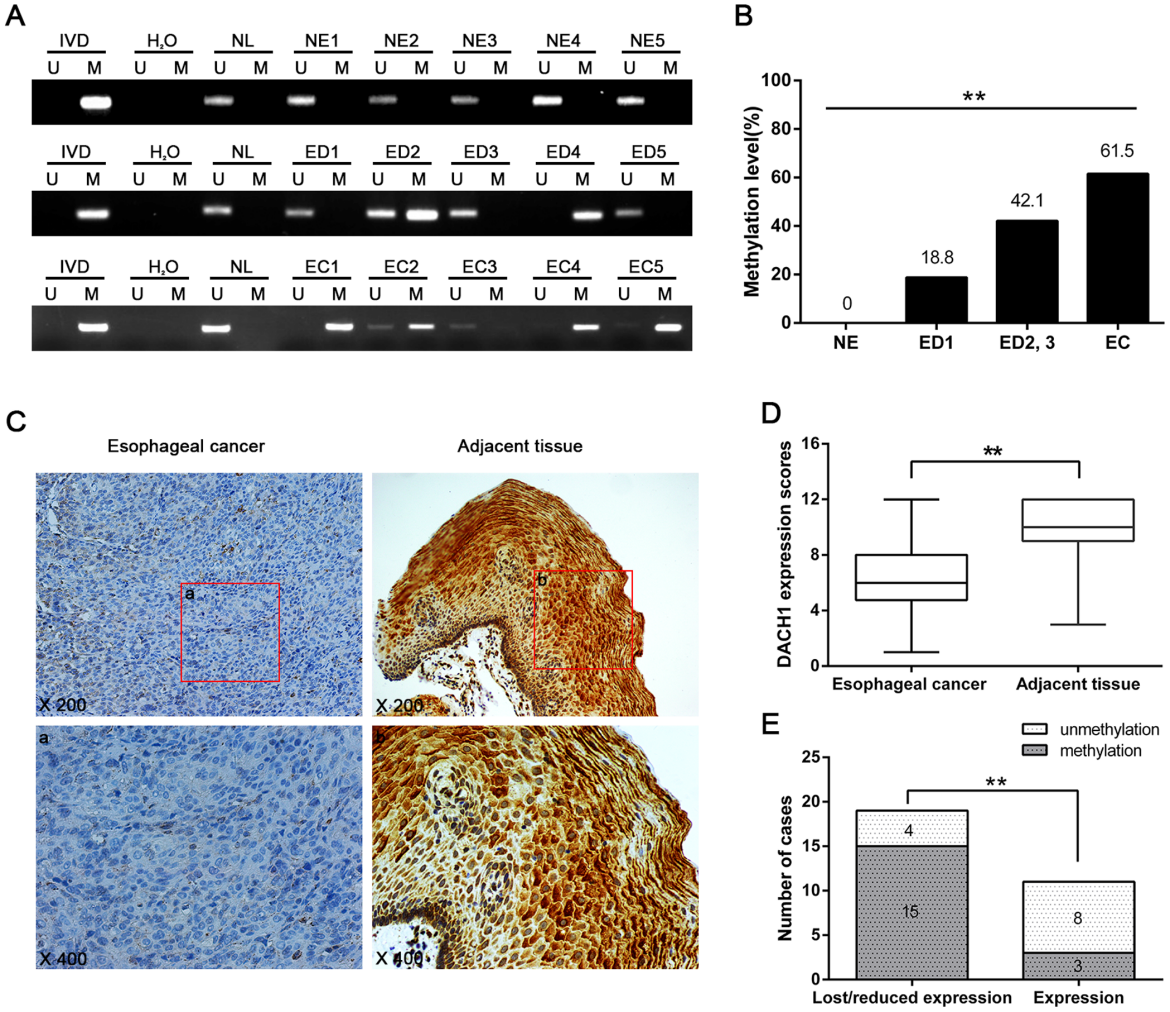
Representative results of *DACH1* methylation and expression in primary esophageal cancer. (A) Representative MSP results of *DACH1* methylation status in normal esophageal mucosa (NE), esophageal dysplasia (ED) and esophageal cancer (EC). (B) *DACH1* methylation frequency in NE, ED1, ED2 and ED3, and EC. The frequency of methylated *DACH1* were plotted according to histological grade and analyzed using chi-square test. **, *P*<0.01. (C) Representative IHC results for DACH1 expression in primary esophageal cancer (left) and adjacent tissues (right); upper phase, X200; lower phase, X400. (D) DACH1 expression level in 30 cases matched primary cancer and adjacent tissue samples; box plot: represents DACH1 expression level; horizontal line: represent the median level; the top and bottom line of the boxes represent 75% and 25% expression level, respectively; vertical bars represent different expression level. **, *P*<0.01 versus adjacent tissue samples by using Wilcoxon signed-rank test. (E) The association of *DACH1* methylation and loss/reduced expression in 30 cases ESCC. **, *P*<0.01, Spearman’s rank correlation coefficient.

To further explore the methylation status of *DACH1* during human ESCC development, 10 cases of normal esophageal mucosa, 51 cases of different grades of dysplasia and 104 cases of primary ESCC were detected by MSP. As shown in figure 2A and 2B, 18.8% (6 of 32) of esophageal grade 1 dysplasia (ED1), 42.1% (8 of 19) of grade 2 and grade 3 dysplasia (ED2,3), and 61.5% (64 of 104) of invasive esophageal cancer (EC) were methylated, but none of the 10 cases of normal esophageal mucosa was methylated. The frequency of *DACH1* methylation was increased in progression tendency during esophageal carcinogenesis (*P*<0.01). *DACH1* methylation was associated with poor differentiation (*P*<0.05) and late tumor stage (*P*<0.05) significantly. No association was found between *DACH1* methylation and age, gender, metastasis or tumor size (*P*>0.05, Table 1). Above results indicate that *DACH1* methylation is an early event of esophageal carcinogenesis and methylation of *DACH1* is accumulated during progression of esophageal cancer. The expression of DACH1 was evaluated by immunohistochemistry (IHC) in 30 cases of matched esophageal cancer and adjacent tissue samples. The expression was reduced significantly in cancer samples (*P*<0.01, Fig. 2C and 2D). It suggest that *DACH1* is a possible tumor suppressor in esophageal cancer. To see if DACH1 expression was regulated by DNA methylation in human primary cancer, the association of DACH1 expression and promoter region hypermethylation was analyzed. Reduced expression was found in 19 cases of cancer tissue and 15 cases were methylated (Fig. 2E). Reduction of DACH1 expression was associated with promoter region hypermethylation significantly (*P*<0.01). These results suggest that DACH1 expression is regulated by promoter region hypermethylation in human primary ESCC.

**Table 1:** Clinicopathologic characteristics and DACH1 methylation status of 104 patients with esophageal squamous cell carcimoma.

		Methylation status		
Clinical parameter	No.	Methylated	Unmethylated	*P* value*
		n = 64 (61.5%)	n = 40 (38.5%)	
***Age (year)***				
<50	27	14 (51.9%)	13 (48.1%)	*P* = 0.2292
≥50	77	50 (64.9%)	27 (35.1%)	
***Gender***				
Male	82	47 (57.3%)	35 (42.7%)	*P = *0.0876
Female	22	17 (77.3%)	5 (22.7%)	
***Differentiation***				
Poorly	45	34 (75.6%)	11 (24.4%)	*P = *0.0103#
Moderately/Well	59	30 (50.9%)	29 (49.1%)	
***Tumor stage***				
I/II	71	39 (54.9%)	32 (45.1%)	*P = *0.0422#
III/IV	33	25 (75.8%)	8 (24.2%)	
***Metastasis***				
Negative	69	39 (56.5%)	30 (43.5%)	*P = *0.1398
Positive	35	25 (71.4%)	10 (28.6%)	
***Tumor size***				
*≥5 cm*	33	18 (54.6%)	15 (45.4%)	*P* = 0.3176
*<5 cm*	71	46 (64.8%)	25 (35.2%)	

*P values are obtained from chi-square test.

# Statistical significance is indicated by *P*<0.05.

### Restoration of DACH1 Expression Suppresses Esophageal Cancer Growth both *in vitro* and *in vivo*

**Figure 3:**
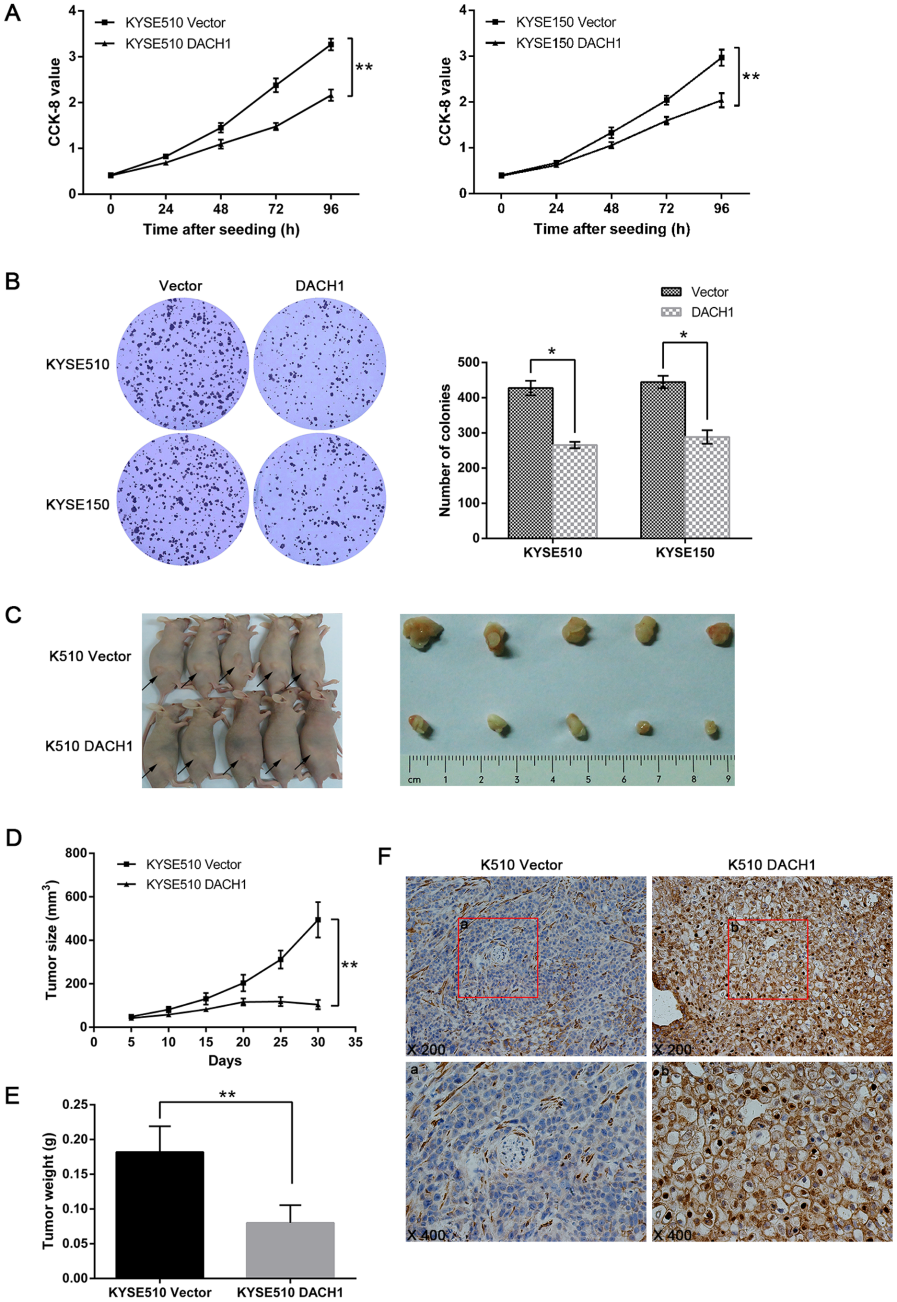
Representative results of *DACH1* suppresses esophageal cancer growth *in vitro* and *in vivo*. (A) Growth curves represent CCK-8 assay results for *DACH1* expressed cells and unexpressed cells.Points, mean of four independent experiments; bars, SEM. **, *P*<0.01, Student’s *t* test. (B) Representative results of colony formation in *DACH1* expressed and unexpressed KYSE510 and KYSE150 cell lines. Columns, mean of four independent experiments; bars, SEM. *, *P*<0.05 versus controls by using the Student’s *t* test. (C) Representatives results of xenograft tumors in nude mice for *DACH1* expressed and unexpressed KYSE510 cells. (D) Growth curves represent tumor size in *DACH1* expressed and unexpressed KYSE510 cells xenograft mice in different time. Points, mean of 5 mice; bars, SEM.*, *P*<0.01, Student’s *t* test. (E) Representative results of tumor weight in *DACH1* expressed and unexpressed KYSE510 cells xenograft mice in different time. Columns, mean of 5 mice; bars, SEM. *, *P*<0.01, Student’s *t* test. (F) Representive DACH1 expression results detected by IHC for *DACH1* expressed and unexpressed KYSE510 cells xenograft. DACH1 expression was found in *DACH1* expressed KYSE510 cell xenograft. (right). Magnification: upper phase, X200; lower phase, X400.

To see the effect of *DACH1* on ESCC cell proliferation, KYSE510 and KYSE150 cells with stably expressing *DACH1* or empty vector were established by lentivirus transduction. Cell viability and colony formation were analyzed in *DACH1* stably expressed cells and unexpressed control. Re-expression of *DACH1* inhibited cell proliferation (*P*<0.01, Fig. 3A) and colony formation (*P*<0.05, Fig. 3B) in these cell lines. The function of *DACH1* on esophageal cancer was also studied in xenograft mice model (Fig. 3C). The tumor size was smaller in *DACH1* expressed KYSE510 cells than in control (104.23±21.38 mm^3^ vs 494.65±81.98 mm^3^, *P*<0.01, Fig. 3D), and the tumor weight was less in *DACH1* expressed KYSE510 cells than in control group (78±28 mg vs 182±37 mg, *P*<0.01, Fig. 3E). The expression of DACH1 was validated by IHC in xenograft (Fig. 3F). These results suggest that *DACH1* suppresses esophageal cancer growth both *in vitro* and *in vivo*.

### Restoration of DACH1 Expression Increased G_1_ Phase and Reduced S Phase Cells

**Figure 4:**
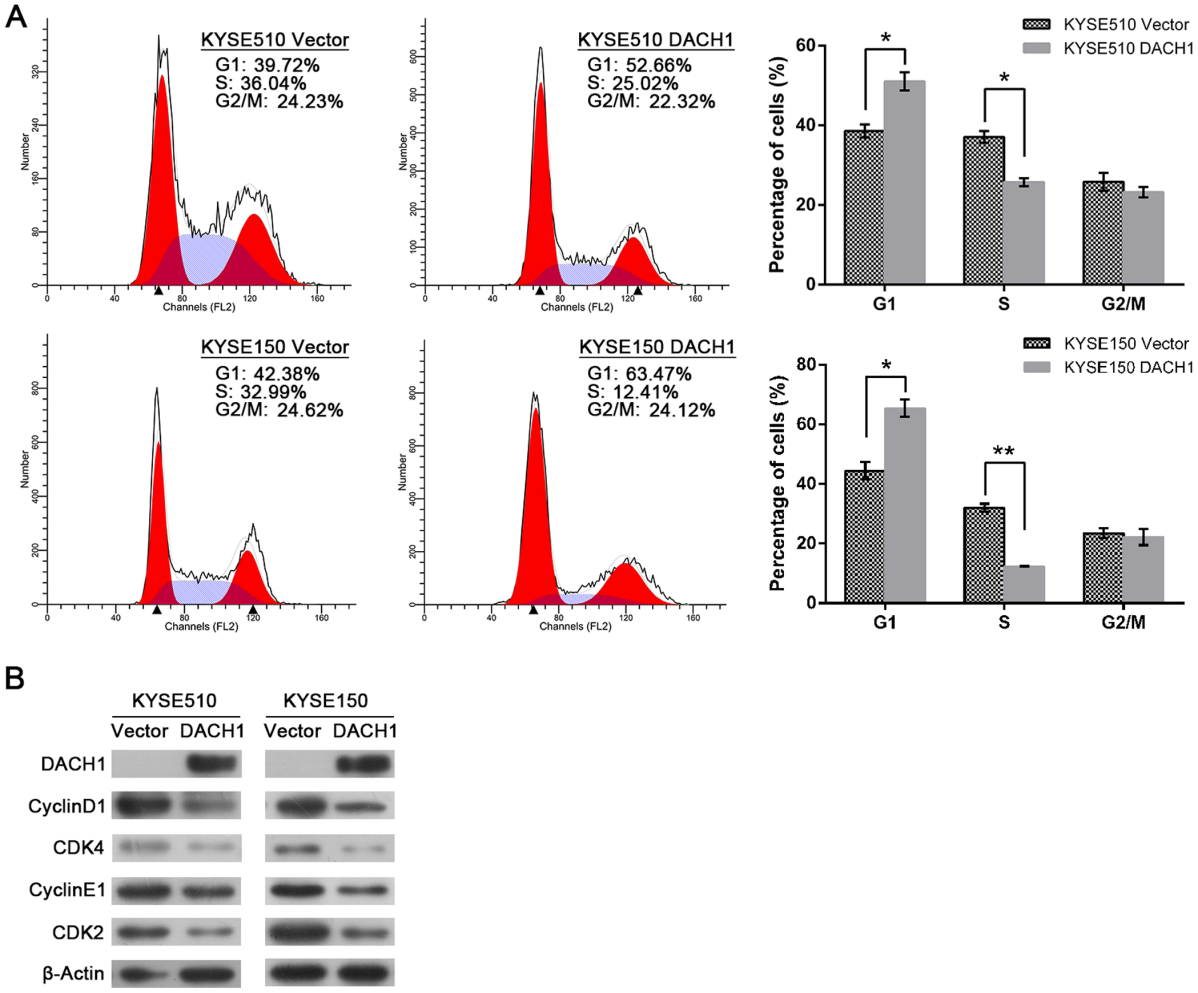
Flow cytometry and western blot results. (A) Flow cytometry results show: the cell phase distribution in DACH1 unexpressed and expressed KYSE510 and KYSE150 cells. Columns, mean of three independent experiments; bars, SEM. *, *P*<0.05; **, *P*<0.01, Student’s *t* test. (B) Western blot results show: expression of G1/S check point related genes in DACH1 unexpressed and expressed KYSE510 and KYSE150 cells, β-Actin was used as a loading control.

The effect of *DACH1* on cell cycle was analyzed by flow cytometry in KYSE510 and KYSE150 cell lines. As shown in figure 4A, The ratio of G1 phase cell was 51.05±2.28% and 38.56±1.64% in *DACH1* expressed and unexpressed KYSE510 cell lines (*P*<0.05). The ratio of S phase was 25.72±0.99% and 37.09±1.49% in *DACH1* expressed and unexpressed KYSE510 cell lines (*P*<0.05). The ratio of G1 phase cell was 65.46±2.84% and 44.41±2.87% in *DACH1* expressed and unexpressed KYSE150 cell lines (*P*<0.05). The ratio of S phase was 12.34±0.10% and 32.06±1.32% in *DACH1* expressed and unexpressed KYSE150 cell lines (*P*<0.01). These results suggest that *DACH1* increases G1 phase and reduced S phase cells in esophageal cancer.

Increased expression of CDK2, CDK 4, cyclinD1 and cyclinE1 usually represents promoting cell cycle from the G1 phase to S phase. As shown in figure 4B, the expression of CDK2, CDK 4, cyclinD1 and cyclinE1 were reduced apparently in *DACH1* expressed KYSE510 and KYSE150 cells compared with unexpressed cells. It suggests that *DACH1* suppresses cell proliferation by inhibiting G_1_/S checkpoint in esophageal cancer. The effect of *DACH1* on cell apoptosis was also analyzed by flow cytometry in KYSE510 and KYSE150 cell lines, but no apoptosis change was found before and after restoration of *DACH1* expression in these two cell lines (data not shown).

### Restoration of DACH1 Expression Activates TGF-β Signaling in ESCC

**Figure 5:**
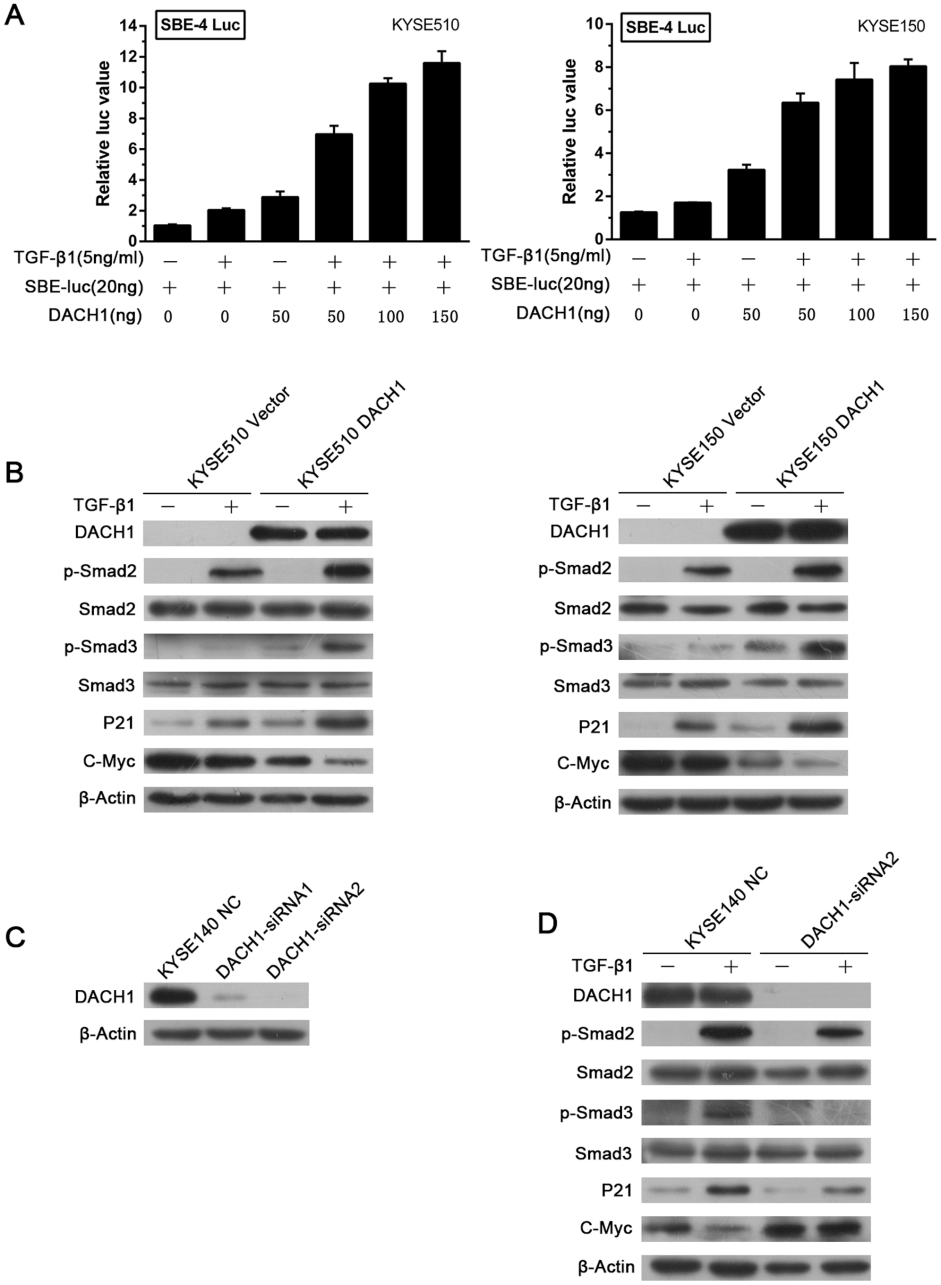
Effect of *DACH1* on TGF-β signaling in human esophageal cancer cells. (A) Smad-binding elements (SBE)-4 Luc reporter activities in KYSE510 and KYSE150 cells. Columns, mean of three independent experiments; bars, SEM. (B) The expression level of TGF-β signaling downstream genes in *DACH1* expressed cells and unexpressed cells, β-Actin was used as a loading control. (C) The efficiency of siRNAs targeting on *DACH1* in KYSE140 cells. (D) The expression level of TGF-β signaling downstream genes in *DACH1*-siRNA KYSE140 cells and control group, β-Actin was used as a loading control.

Our previous study found that *DACH1* performed anti-proliferation effect by activating TGF-β signaling and inhibiting c-Myc expression in human hepatocellular carcinoma cell lines. [13] To determine whether the TGF-β signaling is regulated by *DACH1* in ESCC, dual-luciferase reporter assay was employed to examine SBE4 luciferase activity in KYSE510 and KYSE150 cell lines. As shown in figure 5A, SBE4 promoter activity was increased more than 3 fold in KYSE510 and 2.6 fold in KYSE150 cells after restoration of *DACH1* expression, and the activity was increased in a dose-dependent manner by restoration of *DACH1* expression combined with TGF-β1 treatment. To further understand the mechanism of *DACH1* on TGF-β signaling, the level of phosphorylated Smad2 (p-Smad2) and phosphorylated Smad3 (p-Smad3), and its downstream targets, p21 and c-Myc were evaluated in *DACH1* unexpressed and expressed KYSE510 and KYSE150 cell lines. The level of p-Smad2 was not changed before and after re-expression of *DACH1*, while the level of p-Smad3 was increased after re-expression of *DACH1*. The level of p-Smad2 and p-Smad3 were increased after adding TGF-β1. p-Smad3 was increased apparently when added TGF-β1 to *DACH1* re-expressed KYSE510 and KYSE150 cells. The expression of downstream genes were different. p21 was up-regulated and c-Myc was down-regulated after re-expression of *DACH1* (Fig. 5B). It hints that TGF-β signaling is activated by *DACH1* and TGF-β1 enhances this effect. To further validate the effect of *DACH1* on TGF-β signaling, siRNA knockdown technique was employed. The level of p-Smad2 didn’t change after knocking down *DACH1* in *DACH1* expressed KYSE140 cells, but the level of p-Smad3 was reduced when knocking down *DACH1*. Both p-Smad2 and p-Smad3 were increased after adding TGF-β1. p-Smad3 was increased slightly by adding TGF-β1 to siRNA transfected KYSE140 cells compared with only knocking down *DACH1*. Reduced p21 and increased c-Myc expression were found after knocking down *DACH1* in KYSE140 cells (Fig. 5C and 5D). Above results further suggest that TGF-β signaling is activated by *DACH1* in human esophageal cancer.

## Discussion

The expression of DACH1 was reduced in breast, prostate, lung, endometrial, colorectal and hepatocellular carcinoma, but it was increased in ovarian cancer. [7]–[13], [21] DACH1 expression was regulated by promoter region hypermethylation in endometrial, colorectal and hepatocellular carcinoma. [11]–[13] In the present study, we demonstrated that DACH1 expression was reduced and the expression of DACH1 was regulated by promoter region hypermethylation in human esophageal cancer. We had reported that many tumor suppressors were methylated with a progression tendency during esophageal carcinogenesis. [15], [16], [22], [23] In this study, we analyzed the methylation status of *DACH1* in normal esophageal mucosa, different grades of dysplasia and invasive cancer. *DACH1* was frequently methylated in esophageal cancer and the frequency was increased with the progression of esophageal carcinogenesis from normal esophageal mucosa to invasive cancer. It suggests that *DACH1* is an esophageal cancer early detection marker. The association of poor differentiation and late tumor stage with *DACH1* methylation suggests that *DACH1* methylation may serve as esophageal cancer prognostic marker.

*DACH1* was regarded as a tumor suppressor or an oncogene in different kind of cancer. [7]–[13], [21] In our study, *DACH1* was found to suppress esophageal cancer growth both *in vitro* and *in vivo*. The TGF-β superfamily is a set of multifunctional cytokines that regulate cell growth, differentiation, apoptosis, migration and angiogenesis. [24]–[27] In normal epithelial cells, the TGF-β signaling involves in transcriptional activation of the cyclin-dependent kinase inhibitor p21Cip1, and repression of the growth-promoting transcription factor c-Myc. Cooperatively, these gene responses mediate cell cycle arrest at G1 phase. [28]–[30] TGF-β signaling plays a critical but paradox role in different cancers [31]–[33]. In breast cancer, TGF-β signaling suppresses cell growth in the early stage and promote cancer invasion in the late stage. [34] Previous study in breast cancer showed that DACH1 inhibited TGF-β signaling through binding Smad4. [20] While in hepatocellular cancer, we found DACH1 activated TGF-β signaling by increasing p-Smad3. It enhanced repression of c-Myc expression and cell proliferation. [13].

In support of previous report that DACH1 induced p21 protein abundance and antagonized Myc-induced oncogenic phenotype in breast cancer, [35] we found here that ectopic expression of DACH1 alone in esophageal cancer cells increased p21 and decreased c-Myc protein level (Fig. 5B). Moreover, DACH1 synergized with TGF-β to enhance induction of p21 and repression of c-Myc; correspondingly, knocking down DACH1 suppressed TGF-β signaling in KYSE140 cells. TGF-β signaling can crosstalk with many other pathways. p53 and smads physically interacted and synergically coregulated TGF-β target genes such as p21 and p15. [36] Recent studies demonstrated that DACH1 associated with p53 and enhanced p53 function to induce apoptosis and inhibit tumor growth. Further study showed that DACH1 shared occupancy of −15% p53-bound genes in ChIP sequencing. [7], [9] As DACH1 activated TGF-β signaling (Fig. 5A) and induced phosphorylation of smad2/3 (Fig. 5B), a possible explanation is the activation and stabilization of smad2/3 protein complex by DACH1 like p53. However, detail mechanism needs to be proved.

In conclusion, *DACH1* is frequently methylated in human esophageal cancer and methylation of *DACH1* is involved in the early stage of esophageal carcinogenesis. DACH1 expression is regulated by promoter region hypermethylation. *DACH1* suppresses esophageal cancer growth by activating TGF-β signaling.
